# A Case of Myopathic Dysphagia Secondary to Thyrotoxicosis

**DOI:** 10.1016/j.aed.2025.10.020

**Published:** 2025-11-19

**Authors:** Hsieh En Chua, Lip Hong Tan, Chee Kian Chew

**Affiliations:** 1 Department of Endocrinology, Tan Tock Seng Hospital, Singapore, Singapore; 2 Department of General Medicine, Tan Tock Seng Hospital, Singapore, Singapore

**Keywords:** myopathic dysphagia, dysphagia in thyrotoxicosis, bulbar weakness in thyrotoxicosis

## Abstract

**Background/Objective:**

Myopathic dysphagia is a rare manifestation of thyrotoxicosis. Dysphagia may be isolated or may be associated with preceding proximal myopathy.

**Case Report:**

We describe a 70-year-old man with newly diagnosed Graves’ disease who presented with acute dysphagia with both liquids and solids for 3 weeks, with free thyroxine >73 pmol/L (reference range 8-16 pmol/L), free triiodothyronine >40 pmol/L (reference range 3.5-6.0 pmol/L), thyroid-stimulating hormone <0.01 mIU/L (reference range 0.45-4.50 mIU/L), and thyroid-stimulating hormone receptor antibody 28.6 IU/L (reference range 0.0-1.0 IU/L). This was associated with heat intolerance, palpitations, diarrhea, proximal weakness, and weight loss.

A video fluoroscopy study confirmed moderate oropharyngeal dysphagia. Evaluation was done to exclude other causes including mechanical compression, neuromuscular causes, and malignancy.

The patient’s dysphagia improved with carbimazole doses of up to 30 mg twice daily, and propranolol doses of up to 20 mg 3 times daily, titrated as thyrotoxicosis improved. He became euthyroid after 5 weeks of treatment, and had clinical improvement in swallowing by 8.5 weeks, improving from requiring nasogastric tube feeding to tolerating regular diet and thin fluids.

**Discussion:**

We discuss differential diagnosis to consider and exclude, and the pathophysiology involved in myopathic dysphagia. Based on case reports in literature, patients responded well with high doses of thionamides and beta-blockers.

**Conclusion:**

Our patient’s dysphagia resolved rapidly with normalization of free thyroxine and free triiodothyronine. It is important to render the patient euthyroid as soon as possible to minimize risks of aspiration, pneumonia, and other complications.

**Highlights**

• Myopathic dysphagia is a rare manifestation of thyrotoxicosis, but an important differential diagnosis to consider for dysphagia
• Dysphagia in myopathic dysphagia resolves rapidly with treatment; rendering the patient euthyroid as soon as possible will help minimize risks of complications
• High-dose thionamides along with beta-blockers are effective treatments for myopathic dysphagia
• We present a patient who had significant improvement of his dysphagia after 8 weeks of treatment

**Clinical Relevance**

There is a lack of literature available on management and treatment of myopathic dysphagia. If not promptly treated, complications from myopathic dysphagia may lead to high morbidity and mortality. We would like to share our clinical experience from managing this case.

## Introduction

Thyrotoxic myopathy can present as acute or chronic thyrotoxic myopathy. Acute thyrotoxic myopathy has a rapid onset and may present with bulbar paralysis, whereas chronic thyrotoxic myopathy typically has an insidious onset with slow progression and presents with muscle weakness.^1^^,^^2^

Myopathic dysphagia is a rare manifestation of thyrotoxicosis, which may be associated with proximal skeletal muscle weakness.^3^ Patients may present with dysphagia to both solids and liquids, and both oropharyngeal and esophageal dysphagia have been reported,^3^ with a higher incidence of oropharyngeal dysphagia. In some cases, the onset of dysphagia can be acute.^4^

The median age of patients with myopathic dysphagia from thyrotoxicosis is around 65 years, with higher incidence in females than males.^3^

Another cause of dysphagia is hypokalemic periodic paralysis^5^—in this condition, there are episodes of hypokalemia and paralysis, precipitated by various factors including high carbohydrate intake, strenuous activity and alcohol use. Other causes include external compression from a goiter, neuromuscular etiologies including myasthenia gravis (which patients with Graves’ disease may concurrently have)^2^^,^^6^ and malignancy.

For myopathic dysphagia, prompt treatment of thyrotoxicosis should be commenced to reduce risks of aspiration pneumonia, and morbidity and mortality.^3^^,^^4^^,^^7^

## Case Report

### History

A 70-year-old Chinese man presented with a 3-week history of thyrotoxicosis with heat intolerance, palpitations, diarrhea, proximal muscle weakness, dysphagia, reduced appetite, and significant weight loss—from 67 to 56 kg in a month. His past medical history included well-controlled hypertension, hyperlipidemia, type 2 diabetes mellitus, and stable anemia with history of inactive cold agglutinin disease. His long-term medications were amlodipine, valsartan, atorvastatin (10 mg daily), fenofibrate (300 mg daily), metformin, and folic acid.

He had consulted a family physician 3 days prior to admission. A thyroid function test was done. Free thyroxine (fT4) was >83.0 pmol/L (reference range 12.0-22.0 pmol/L) and thyroid-stimulating hormone was <0.005 mIU/L (reference range 0.270-4.200 mIU/L). He was subsequently started on carbimazole 10 mg daily. However, his symptoms persisted, accompanied by progressive functional decline, which prompted his admission to the hospital. Atorvastatin and fenofibrate were held on admission.

He reported an acute onset of dysphagia with both solids and liquids, which developed concurrently with proximal muscle weakness. He did not notice any neck swelling or palpable masses. There were no preceding symptoms of fever, upper respiratory illness, or neck tenderness to suggest thyroiditis. He also denied any recent exposure to excess iodine including contrast administration or amiodarone. He had a first-degree relative with a history of neck swelling who underwent thyroid surgery at age 50s, although our patient was uncertain about the diagnosis.

His carbimazole dose was increased on admission and was titrated based on the trend of his thyroid function tests (Table 1). A diagnosis of Graves’ disease was made when the thyroid-stimulating hormone receptor antibody returned strongly positive (Table 1). Electrolytes were all unremarkable, with no hypokalemia to suggest hypokalemic periodic paralysis. His potassium was 4.6 mmol/L and hemoglobulin was 11.0 g/dL on initial review.

**Table 1:** Thyroid Investigations and Carbimazole Dose Titration

Abbreviations: fT4 = free thyroxine; fT3 = free triiodothyronine; TRAb = TSH receptor antibody; TSH = thyroid-stimulating hormone.

This table trends the thyroid function tests done and corresponding carbimazole dose titration. Total T3 was not trended as the test turnaround time in our institution then was around 3 days.

### Physical Examination

On our initial review, Mr. Y. was alert, afebrile, saturating well on room air, hemodynamically stable, and intermittently mildly tachycardic with heart rate of up to 110 beats per minute. Heart sounds were dual and regular in rhythm on examination. He had a lean habitus with a body mass index of 19.7 kg/m^2^.

He had no thyroid eye disease, lid lag, or pretibial myxedema. He had no palpable goiter or thyroid nodule, cervical lymphadenopathy, or neck tenderness. He had no hand tremors. Clinically, he had no features of thyroid storm—he was not febrile or jaundiced, he was euvolemic with no signs of congestive cardiac failure, and his abdomen was soft and nontender. Burch-Wartofsky Point Scale for thyrotoxicosis was 20 points (10 points heart rate, 10 points for diarrhea).

On neurological examination, there were no tongue or limb fasciculations, ophthalmoplegia, diplopia, fatigability, or signs of Parkinsonism. Medical Research Council scale power of bilateral hip flexion was 4, but otherwise power was full for: neck flexion and extension, bilateral upper limb proximal and distal power, bilateral hip extension and rest of lower limb power. Bilateral upper limb and lower limb reflexes were brisk, and sensation was intact on testing of fine touch over all limbs.

### Treatment

Thyroid investigations done, along with carbimazole dose titration during the admission, are as shown in Table 1.

### Multidisciplinary Input and Further Evaluation

His dysphagia led to recurrent aspiration pneumonia approximately a week into admission, which was subsequently complicated by a type 2 myocardial infarction, for which atorvastatin 40 mg was initiated end of November 2024. Following assessment by the speech therapist, oral feeding was deemed unsafe and nasogastric tube feeding was commenced.

The patient was reviewed by a multidisciplinary team to further evaluate his dysphagia. The otolaryngology specialist performed a nasal endoscopy, which did not reveal any masses. A video fluoroscopy (VFS) demonstrated moderate oral pharyngeal dysphagia and esophageal hold up. He was reviewed by a gastroenterologist. A computed tomography scan of the thorax, abdomen, and pelvis did not reveal any finding suggestive of malignancy. He had a recent esophagogastroduodenoscopy and colonoscopy performed 9 months prior to admission which did not reveal any mass or malignancy. A barium swallow was initially planned but later deferred as his swallowing had already improved significantly.

Magnetic resonance imaging of the brain did not reveal any infarcts, intracranial hemorrhage, or demyelinating lesions. He was subsequently reviewed by a neurologist, and further evaluation was done (Table 2) to rule out other neurologic causes.

**Table 2:** Neurologic Investigations

Test	Result	Reference range and units (if applicable)
Acetylcholine receptor antibodies	Negative	
Antimuscle specific kinase antibodies	Negative	
Anti-Mi-2a antibody	Negative	
Anti-Mi-2b antibody	Negative	
Anti-TIF1g antibody	Negative	
Anti-MDA5 antibody	Negative	
Anti-NXP2 antibody	Negative	
Anti-SAE1 antibody	Negative	
Anti-Ku antibody	Negative	
Anti-OJ antibody	Negative	
Anti-Ro-52 antibody	Negative	
Anti-cN-1A antibody	Negative	
Anti-HMGCR antibody	Negative	
Aldolase	4	0-8 U/L
Lactate dehydrogenase	430	270-550 U/L
Creatine kinase	33	50-350 /L

Nerve conduction test and electromyography (EMG) done showed electrodiagnostic evidence of a nonirritable myopathy involving proximal muscles in both upper and lower limbs, in keeping with thyrotoxic myopathy. Repetitive nerve stimulation confirmed no postsynaptic neuromuscular junction pathology.

Creatine kinase, lactate dehydrogenase, and aldolase levels may be normal or decreased in thyrotoxic myopathy.^1^^,^^8^^,^^9^ EMG is important for diagnosis as up to 32%^10^ do not show clinical myopathy despite positive findings on EMG.

### Diagnosis

In conclusion, he was diagnosed with myopathic dysphagia from thyrotoxicosis with underlying newly diagnosed Graves’ disease. He had acute thyrotoxic myopathy evidenced by his acute onset of symptoms with rapid progression of dysphagia, which was complicated by aspiration pneumonia.

### Progress

While his hyperthyroidism was being treated, he also underwent swallowing therapy with the speech therapists. Repeat VFS examination was done 8.5 weeks after admission, which showed mild pharyngeal dysphagia with reduced hyolaryngeal excursion, and the speech therapist recommended to upgrade his diet to regular diet and thin fluids with independent feeding as his swallowing had improved significantly. He tolerated these diet consistencies thereafter and remained well. On outpatient clinic review in August 2025, Mr Y. was clinically euthyroid with normal fT4 level and has had no further episodes of dysphagia. Definitive treatment of his underlying Graves’ disease has been discussed but he remains undecided for now.

## Discussion

The pharyngeal muscles and upper third of the esophagus are made out of skeletal muscles,^11^ which are affected in thyrotoxicosis. The mechanism of myopathic dysphagia is not clear; however, it is theorized that high thyroid hormones can affect neuromuscular transmission,^12^ muscle myogenesis and contractile function,^11^^,^^13^ cause muscle degradation^14^ (thus muscle enzymes levels such as creatine kinase may be normal or decreased,^1^^,^^8^^,^^9^ as compared with in hypothyroidism), and increase basal metabolic rate and cause mitochondrial dysfunction.^15^ Glycosaminoglycan deposition may also cause interstitial edema in esophageal muscles.^16^ A study done by Karaman et al^17^ using esophageal manometry showed that the duration of esophageal muscle contraction was significantly shorter in hyperthyroid patients.

In literature (Table 3),^4^^,^^7^^,^^11^^,^^12^^,^^16^^,^^18^, ^19^, ^20^ some physicians recommend treating myopathic dysphagia with high doses of antithyroid drugs, such as those used in thyroid storm, to render the patient euthyroid as quickly as possible.^3^ This helps to reduce risks of aspiration pneumonia, and morbidity and mortality. Antithyroid drugs lower synthesis of thyroid hormones by inhibiting thyroid peroxidase and hence oxidation of iodide to iodine, organification, and coupling of monoiodotyrosine and diiodotyrosine.^21^ Antithyroid drugs also have an immunosuppressive effect.^22^ Beta-blockers such as propranolol reduce peripheral conversion of fT4 to free triiodothyronine, reducing sympathetic activity and beta-2 adrenergic stimulation in myocytes,^23^ and have been shown to be effective in treatment of myopathic dysphagia.^12^^,^^16^^,^^24^

**Table 3:** Case Reports in Literature

Case reports	Case details	Time to symptom improvement or resolution
Andrews et al,^11^ 2025	• 41-y-old woman with Graves’ disease • Dysphagia to liquids and solids, nausea, vomiting, diarrhea, 30 lb weight loss • Treated with methimazole, propranolol, hydrocortisone • Underwent OGD and CT imaging • Due to the severity of the patient's symptoms, worsening nutritional status, intolerance to oral or rectal formulations of standard medical therapy, and lack of surgical candidacy, decision for plasmapheresis was made • 3 sessions of plasmapheresis performed on alternate days	Dysphagia was reversed completely within 24 h after 1 plasmapheresis session
Mathialagan et al,^16^ 2024	• 44-y-old male with Graves’ disease • Dysphagia to solids, weight loss, and dyspepsia for 6 wk • CT neck was unremarkable, no nodules on thyroid ultrasound • Modified barium swallow showed abnormal oral and pharyngeal swallow phase with increased residue • Treated with methimazole and propranolol	Symptoms improved after 2 mo
Mathew et al,^18^ 2011	• 52-y-old male with Graves’ disease • Severe dysphagia, dysphonia, complicated by aspiration • Isolated acute bulbar palsy without skeletal muscle involvement	Symptoms recovered within 6 wk
Parperis et al,^12^ 2011	• 82-y-old male with Graves’ disease • 4-wk history of dysphagia and weight loss of 25 lb, generalized weakness • Work up done included upper endoscopy, MRI brain, EMG, acetyl-cholinesterase receptor antibodies and voltage-gated calcium channel antibodies (negative). Modified barium swallow test showed oropharyngeal dysphagia. • Patient was treated with methimazole 10 mg 3 times daily and propranolol 20 mg every 6 h	Tolerated liquids and soft diet 5 d later, symptoms completely resolved 4 wk after
Okada and Yoshioka,^7^ 2009	• 36-y-old male • Presented with thyroid storm, dysphagia, aspiration pneumonia • Treatment: antithyroid agent, beta-blocker, potassium iodide and glucocorticoid	Dysphagia resolved after 2 mo
Chiu et al,^4^ 2004	• 3 cases • Antecedent muscle weakness before the onset of dysphagia, or sudden onset of bulbar palsy • Either a myopathic or neuropathic pattern was found on electromyography. The incidence of oropharyngeal dysphagia was higher than that of esophageal motility dysfunction. • Aspiration pneumonia occurred more accompanied by oropharyngeal dysphagia • Suggested for early intensive treatment that includes antithyroid agent, beta-blocker and Lugol solution	Resolved within 3 wk
Ramanathan,^19^ 1989	• 51-y-old male • Dysphagia for 3 mo with both liquids and solids, loss of appetite and loss of weight, associated with vomiting • Had proximal myopathy • Normal endoscopic and barium meal examinations • Treated with propylthiouracil 150 mg 3 times daily and propranolol 40 mg twice daily	Documented that symptoms resolved rapidly once euthyroid
Sweatman and Chambers,^20^ 1985	• 71-y-old female • Dysphagia for 3 mo, progressive muscle weakness, loss of appetite • EMG: myopathic pattern • Treated with carbimazole 15 mg 3 times daily and dysphagia recovered	Not specified

Abbreviations: CT = computed tomography; EMG = electromyography; MRI = magnetic resonance imaging; OGD = esophagogastroduodenoscopy.

Other thyroid etiologies of dysphagia to consider include hypokalemic periodic paralysis which may present with bulbar symptoms,^25^ and external compression from a goiter. It is also important to exclude neuromuscular causes including myasthenia gravis (which patients with Graves’ disease may concurrently have^2^^,^^6^) and malignancy.

In hypokalemic periodic paralysis, cases may show a myopathic pattern on EMG during paralysis, which resolves during remission^26^—this may be useful to help differentiate the condition from acute thyrotoxic myopathy. Absence of hypokalemia and persistent symptoms in our case did not suggest hypokalemic periodic paralysis.

Our patient did not have any palpable goiter or thyroid nodule, and intrinsic and extrinsic compression were ruled out with nasal endoscopy, VFS, and ultrasound thyroid. A barium swallow was planned to evaluate for luminal narrowing or obstruction but was held off as his swallowing had already improved significantly. A computed tomography scan of the thorax, abdomen and pelvis was done to exclude malignancy, and repeat esophagogastroduodenoscopy and colonoscopy were considered but held off as both had been done 9 months prior. He had no features of other neuromuscular disease including myasthenia gravis—he had no clinical fatigability, and he tested negative for acetylcholine receptor antibodies and anti-muscle-specific kinase antibodies. Magnetic resonance imaging brain and neurological evaluation did not point toward any neurological conditions.

Our patient became euthyroid after 5 weeks of treatment and had clinical improvement in swallowing by 8.5 weeks, improving from requiring nasogastric tube feeding to tolerating regular diet and thin fluids. Our management approach shows that a high dose of antithyroid drug and propranolol of up to 60 mg/d were effective in treating his thyrotoxicosis with fast resolution of his dysphagia. Aggressive treatment is needed to render the patient euthyroid as quickly as possible to prevent complications, morbidity and mortality.

Based on case reports in literature, the median age is around 65 years,^4^ and there are several Asian patients. Our patient was older than the average median age reported in literature.

Further research is needed to understand the mechanisms behind myopathic dysphagia in thyrotoxicosis, and perhaps more prospective studies can be done to support an aggressive treatment approach.

## Conclusion

This case highlights the wide differentials of dysphagia in patients with thyrotoxicosis and the importance of considering myopathic dysphagia, while excluding other causes. High doses of antithyroid drugs (such as doses used in thyroid storm) should be used to treat myopathic dysphagia, to render the patient euthyroid as quickly as possible.^3^ This helps to reduce risks of aspiration pneumonia, and morbidity and mortality. Beta-blockers such as propranolol have been shown to be effective in treatment.^12^^,^^16^^,^^24^

## Disclosure

The authors have no conflicts of interest and no sources of funding to disclose.
